# The Ethical in Modern Medicine

**Published:** 1895-03-02

**Authors:** 


WITH

AN EXTRA NURSING SUPPLEMENT.

VOL. XVII.—No. 440.] [MARCH 2, 1895.

### THE ETHICAL IN MODERN MEDICINE.

WHY do we of the present generation so persistently put forward the phrase and the idea of "modern medicine"? Medicine is medicine, it may be said; and whether it be ancient, or modern, or mediæval, it is still "medicine," and can never be anything else. Such a criticism entirely fails to grasp the real merits of the situation. Medicine is medicine, it is true. But for practical purposes modern medicine is not ancient medicine, any more than the astronomy of the Astronomer Royal of to-day is the astrology of the soothsayers of ancient Chaldea. In purpose, in intention, in method, and as far as is possible in every detail of daily and hourly practice, modern medicine is scientific; scientific in the sense that it is based upon observations, experiments, and facts as exact and precise as it has been possible for human faculties to make them. Such a medicine differs *toto cœlo* from the ancient medicine, which was for all practical purposes based upon the speculations and excogitations of more or less ingenious theorists, who did not investigate the human and animal organisms for their data, but took as their starting points the theories of their predecessors, and as their tests and standards of truth their own supposed superior judgments thereupon.

Here is a change of mental attitude which can only be adequately described as a total right-about-face. Such a change has inevitably carried with it a change of ethical attitude. He who is scientific in physiology and practical medicine cannot but be scientific in ethics too; cannot, that is to say, unless he is either a very phenomenal knave indeed, or a more than ordinarily stupid fool. It is a matter of daily experience that the mind of man is moulded in all its parts by that which is the leading occupation or the ruling passion of its life. The mathematician, for example, gradually becomes as exact and precise in his domestic life, his social behaviour, and even in his religion as he is in his class-room and his study. He cannot help it. The mind working daily and hourly on lines of strict exactitude, gradually, and in course of time, becomes unwilling and even unable to work on any other lines.

A precisely similar case is that of the modern medical man. Working on the lines of careful observation, of truthful recording, and of honest judgment in the consulting-room and at the bedside, he, unconsciously and almost without effort, becomes exactly truthful and judicially honest in every other relation in life. Even men who have not been by nature endowed with any unusual development of moral scrupulosity, acquire after twenty year's competent study and practice of scientific medicine, a plain downrightness and simplicity of mind which cannot tolerate anything but "the thing as it is." We are not now affirming that there is any unusual moral merit in this; we are simply stating a fact which has a most important bearing upon the general thesis which this brief article is written to maintain.

The contention is that we have in modern medicine the elements of an altogether higher and worthier ethic than was possible to our predecessors of the last and preceding centuries. The scientific medical man, we have contended, cannot help being honest, and cannot help a very deep feeling of contempt for those of his fraternity who utilise the arts of the untruthful puffer and the self-advertiser in the furtherance of their advancement. He does not sufficiently recognise that the scientific sense, as it may be called, is not possible for everyone; whilst selfishness and the desire for personal advancement are elementary physiological endowments which are the property of the whole race, and which the race has in common with the lower animals. What we desire to reach as a conclusion is this, that, since we have in modern medicine the necessary means for the development of a higher ethic, we ought to take the necessary steps to organise and concentrate those means, so that they may produce for us the fruits of a higher and more scientific morality throughout the whole trunk and branches of the tree of medical life. In a word, just as it is not reasonable to expect that there shall be the same scientific energy in the daily practice of the medical art at the periphery of our profession as there is at its more highly vitalised centres, so we may not reasonably look for the same scientific elevation of professional morality. But just also as the centre of the professional nervous system is constantly forcing a more scientific medical art to the circumference, so it must consciously and constantly propel a more scientific and a more elevated ethic. It is the centre which must energise and vitalise the circumference, not the circumference the centre. The question, by what practical methods these principles may be carried into serviceable effect, must remain for future consideration.

